# Molecular switch in human diseases-disintegrin and metalloproteinases, ADAM17

**DOI:** 10.18632/aging.203200

**Published:** 2021-06-28

**Authors:** Guang Yang, Mengying Cui, Weibo Jiang, Jiyao Sheng, Yongsheng Yang, Xuewen Zhang

**Affiliations:** 1 Department of Hepatobiliary and Pancreatic Surgery, The Second Hospital of Jilin University, Changchun 130041, P.R. China; 2 Jilin Provincial Key Laboratory on Molecular and Chemical Genetic, The Second Hospital of Jilin University, Changchun 130041, P.R. China; 3 Department of Orthopaedic, The Second Hospital of Jilin University, Changchun 130041, P.R. China

**Keywords:** ADAM17, domain, human diseases, targeted therapy, miRNAs

## Abstract

The ADAMs (a disintegrin and metalloproteinase) are a family of cell surface proteins with crucial roles in the regulation of cell adhesion, cell proliferation to migration, proteolysis and cell signaling transduction pathways. Among these enzymes, the ADAM17 shows significant effects in the “ectodomain shedding” of its substrates such as cytokines (e.g., tumor necrosis factor α, TNFα), growth factors (e.g., epidermal growth factor, EGF), adhesion proteins (e.g., L-selectin), and their receptors (e.g., IL-6R and TNFα). Several studies focus on the underlying molecular mechanisms of ADAM17 in diseased conditions. Here, we took several different approaches to elucidate the function of ADAM17, the participation of ADAM17 in several human diseases, and the potential as targeted therapy reagents. As more and more studies verify the miRNA-mediated expression variation of ADAM17, the specific regulation network of miRNAs and ADAM17 was exploited in this review as well.

## INTRODUCTION

A disintegrin and metalloproteinase (ADAMs) are a family of membrane-anchored proteins with variety functions in multicellular organisms. ADAMs are important in both physiological and pathological processes and becoming promising molecules in targeted therapy [1]. Proteins of the family perform functions including proteolysis, cell adhesion, cell fusion, and cell signaling [1]. Among the 21 members of ADAM family, 13 are active enzymes. Other ADAMs lacked the catalytic site of Zn-binding sequence (HEXXHXXGXXH) or with destroyed metalloenzyme domain, resulting in proteolytically inactive [2]. However, these molecules were important in intracellular cell signal transduction [1].

**Figure 1:**
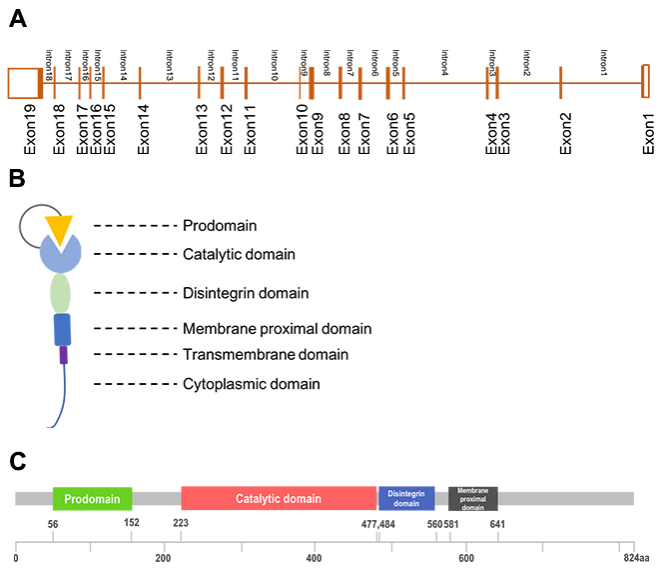
**The structure of ADAM17.** (**A**), Gene structure; (**B**), Generalized domains of ADAM17; (**C**), Conserved domains of ADAM17.

ADAM17 is a member of ADAM family, the ADAM17 gene is located on chromosome 2p25, including 19exons and 18 introns (Figure 1A). Its protein is multi-domain that consist of a prodomain, a metalloenzyme or catalytic domain, a disintegrin domain, a cysteine-rich domain and a transmembrane domain (Figure 1B, 1C). ADAM17 was discovered in 1997 as the enzyme that could proteolysis TNF-α while it was regarded as adhesion proteins in the previous [3, 4]. These two properties enable ADAM17 to participate in cellular adhesion and proteolytic cleavage of various cell surface molecules [1]. Proteolysis is one of the important post-translational modification of transmembrane proteins, while the ADAM17-mediated ectodomain shedding is the main form of proteolysis [5]. More than 10% of all cell surface proteins and most of the transmembrane proteins need to be proteolytically cleaved to release soluble form to be active [6], and the proteolysis usually occurs at the membrane-adjacent part of the molecule [2]. At least 90 substrates were reported to be processed by ADAM17 [7], of which interleukin-6 receptor (IL-6R), the pro-inflammatory cytokine tumor necrosis factor α (TNFα) and the epidermal growth factor receptor (EGFR) were most important [5]. ADAM17 knockout mice develop TGF-α related phenotype such as open eyes and wavy hair at birth, indicating that both TGF-α and TNF-α could be the substrates and cleaved by ADAM17 [8]. Lacking of ADAM17 (ADAM17-null mice) influenced the ADAM17-derived EGFR activation and result in defective valvulogenesis in newborn mice [7, 9]. The proteolytically activated ADAM was localized mainly in the plasma membrane [10]. Protein cleavage can regulate cellular signaling and affect cell behavior, but the results always differs. Both substrate and receptor can be cleaved. The activation of either substrate or receptor can result in different biological functions which partly depend on the pathway the substrate or receptor involved in [11]. ADAMs could be tissue-specific and has preferences for certain proteins [2].

Our review focuses on ADAM17, one of the most well studied ADAM enzymes.

## Functions of different domain of ADAM17

### Prodomain

The prodomain of ADAM17 was the initial inhibitor of the enzyme, and the proteolytic cleavage of its prodomain was necessary for the activation of ADAM17 [12, 13]. Furin, a pro-protein convertase, was responsible for this reaction [14]. Isolated prodomain could inhibit the proteolytic process of ADAMs and acted as effective and selective inhibitors of active ADAMs [15]. Studies showed that deletion of the prodomain damaged the proteolytic activity of the protein [16] and the secretion of TNF-alpha was inhibited after the overexpression of prodomain of ADAM17 [13]. The prodomain may be not necessary for the transportation to cell surface, but it might play important role in the extracellular system [16]. Overexpression of ADAM17 with mutations in pro-, membrane-proximal- and cytoplasmic-domain showed different influences on the function of ADAM17. Of note, mutations in pro-domain resulted in the impaired proteolytic activity and cell membrane transportation, suggesting the crucial role of pro-domain for ADMA17 activation, protein trafficking and proteolysis [17].

### Catalytic domain

The catalytic domain or metalloenzyme domain of ADAM17 contains a catalytic site sequence with three histidine residues (HEXXHXXGXXH) and one glutamic residue, which are responsible for Zn-binding and the cleavage of peptide bonds. There are two highly conserved and adjacent cysteine sulfhydryl motifs (cysteine-X-X-cysteine, CXXC), and the motifs were the targets for the exchange of protein’s thiol-disulfide [18]. The processing of various membrane bound proteins is heavily dependent on the catalytic domain [1]. For example, process the proTNF-alpha to a soluble form. As ADAM17 involved in various inflammatory diseases, the design of inhibitors of ADAM17 are becoming the targets of disease treatment [19]. The inhibition of ADAM17 by tissue inhibitors of metalloproteinases (TIMPs) was studied by the overexpression of catalytic domain of mouse ADAM17 and confirmed that TIPM-3 was the most important TIPM that involved in the regulation of the secretion of TNF-alpha [19]. On the other hand, the effective binding of TIMP-3 with ADAM17 was due to the extension of TIMP-3 to the hydrophobic pocket of ADAM17 surface and the interaction through the binding edge with active-site cleft of ADAM17 [20].

### Disintegrin domain

Integrins are adhesion receptors mediate the interaction of cell-cell and cell-extracellular matrix (ECM), and participate in many cell progressions including cell adhesion, cell migration and proliferation [21]. Integrin is a prognostic indicator and up-regulated in many types of cancers [22]. Disintegrins are a family of small cysteine-rich peptides that could bind to integrins [23]. The disintegrin domain of ADAM17 could competitively inhibit the function of integrin and was first discovered in viper venom [24]. Later, disintegrins was confirmed in not only platelets but endothelial cells [1]. Studies showed that ADAM17 could bind to α5β1 integrin by the founding of the co-localization in Hela cells [25]. The binding of disintegrin and integrins mediate cell adhesion of itself and neighboring cells, activate various receptors and result in the initiation of several cell signaling pathways [21]. In addition, the disintegrin domain of ADAM17 enabled cancer cells to interact with fibroblast and microenvironment, while soluble disintegrin impair this interaction and increased the proteolysis activity of ADAM17 [26].

### Cytoplasmic domain

Function experiments using truncating mutation of ADAM17 suggested that the transmembrane domain was necessary for the cleave of TNF-alpha. Lacking acids from amino-terminal of cytoplasmic domain increased the activity of ADAM17 mediated TNF-alpha shedding [27]. Of note, the cytoplasmic domain was critical for the activation of integrin-disintegrin binding-mediated magnification cascade of signaling pathways and other signaling like focal adhesion kinase (FAK), extracellular regulated kinase (ERK1/2), and protein kinase B (AKT/PKB) [28]. However, most of the activation of these signaling contribute to the progression and drug resistance of cancer treatment [28].

## Post translational modifications of ADAM17

Post translational modifications of ADAM17 including the removal of pro-domain, and glycosylation or phosphorylation of the enzyme. It is not very clear whether there are differences in glycosylation of ADAM17 between normal and cancer cells and so does the relationship of glycosylation and enzymatic activity. Kinetic parameters analysis for hydrolysis of TNFα-based substrates by insect- and mammalian-expressed human ADAM17 showed that glycosylation of ADAM17 can influence the enzyme activity *in vivo* [29]. Furthermore, zinc-binding and non-zinc-binding inhibitor of ADAM17 exhibit different potency, suggesting that glycosylation of ADAM17 may participate in the cell signaling regulation [29]. An experiment using TNFα substrate with and without a glycan moiety attached to test the change of ADAM17 activity and results indicated that glycosylation enhanced ADAM17 activity [30]. N-linked glycosylation sites on different domain ADAM17 also explained the glycosylation can be the important regulator of ADAM17 [31].

As to another important post-translational modification-phosphorylation, including serine and threonine residues of its cytoplasmic domain was confirmed to be related to many diseases. Protein kinase C and kinase G (PKC, PKG) [32], extracellular-signal regulated kinase (ERK) [33], p38-mitogen-activated protein kinase (p38-MAPK) [33, 34], phorbol ester (PMA) [35], Epidermal Growth Factor (EGF) [35] and phosphoinositide dependent kinase 1 (PDK1) [36] were kinases that confirmed to regulate the phosphorylation of ADAM17. The phosphorylation of the cytoplasmic tail of ADAM17 by ERK or p38-MAPK increase ADAM17-mediated proteolysis of TNFα, which associated with the cell surface dimerization [37]. The C-terminal T735 and/or S791 phosphorylation of ADAM17 in gastric epithelial cells induce the activation of ADAM17, while the threonine phosphorylation by p38 MAPK promote the tumorigenic activity of ADAM17, and the proportion of phospho-ADAM17 was highly correlate with KRAS mutation in lung adenocarcinoma [34]. Other studies also demonstrated that PMA and EGF induce the phosphorylation of ADAM17 on T735 and S819 by extracellular signal-regulated kinase [35]. PDK1 and phosphatidylinositol 3-kinase (PI3-K) contribute to the ADAM17 phosphorylation and induce EGF receptor activation which may enhance the therapeutic effects of EGFR inhibitors in non-small cell lung cancer (NSCLS) patients [36]. However, there are controversy results showed that the activation of ADAM17 was independent of the intracellular portion of ADAM17 [38, 39]. Deletion of entire cytoplasmic portion of ADAM17 could shed the TNFα from the cell surface also [40].

## ADAM17-related human diseases

ADAM17 is essential in the maintenance of homeostasis. The dysregulation of ADAM17 involved in various pathological states including inflammation, tumorigenesis, and central nervous system diseases [2].

### Inflammation

ADAM17-dependent cleavage of relevant substrates which are cytokines including TNFα, Interleukin-6, and their receptors-TNF receptors 1 and 2, IL-6 receptor occurred in inflammatory diseases.

Increased TNF release is associated with numerous inflammatory conditions, and the inflammatory process regulated by TNFα was largely attributed to its soluble form that proteolysis from its membrane-bound form by ADAM17 [3]. As a result, it was possible that the diseases with upregulated circulating TNFα accompanied by enhanced ADAM17 activity [41]. This modification was confirmed in rheumatoid arthritis and osteoarthritis as active ADAM17 was detected in synovial and cartilage tissue in patients with these two diseases [41]. In endotoxin-activated macrophages, the TNF release was regulated via lipoprotein receptor-related protein 1 (LRP1) mediated upregulation or downregulation of TIMP-3 (one of the endogenous inhibitors of ADAM17), and the levels of TIMP-3 could be changed following LPS stimulation [42]. All these results indicated that ADAM17 activity must be tightly regulated in inflammatory diseases. On the other hand, TNFα and its two receptors TNFRI and TNFR2 are all substrates of ADAM17 [40]. The signaling pathways via TNFR1 related to apoptosis or cell death while pathways via TNFR2 seems protective [43]. However, different receptors are sensitive to different forms of TNFα, TNFRI is mainly stimulated by soluble TNFα while TNFRII is mainly stimulated by transmembrane TNFα [44, 45]. The ratio of soluble/transmembrane TNFα was regulated by ADAM17, which elucidated one of the mechanisms of ADAM17 in the regulation of immune system [43].

Soluble IL-6R receptor was responsible for the pro-inflammatory process and becoming an attractive therapeutic target. The activation of soluble IL-6R was carried out by the proteolytic cleavage of the IL-6R by ADAM17 [46]. Of note, one of the common characters of inflammation was the IL-6-induced shift of neutrophil to monocyte. However, neutrophil IL-6R shedding by ADAM17 may be the trigger of immune response to inflammation [47]. In addition, the cleavage of other members of interleukin family-IL-15Rα was mediated by ADAM17 as well, fibroblast cells with deficient ADAM17 usually accompanied with downregulated soluble IL-15Rα [48].

The molecular mechanism of ADAM17 participate in the inflammatory procedures was explored in several other different studies. ADAM activation was required for lymphocytes transfer across the high endothelial venules to lymph nodes which was the basis for mature dendritic cells initiate immune response [49]. Another immune cell-neutrophil was regulated by ADAM17 in the inflammatory response, ADAM-17 dependent L-selecting shedding downregulated its expression and directed neutrophils to inflammatory sites [18]. Reduction-oxidation reaction in disintegrin/cysteine-rich region of ADAM17 may be the mechanism of neutrophil-related L-selectin shedding [18]. Molecules took part in the activation of leukocyte such as vascular cell adhesion molecule, intercellular adhesion molecule-1 could be cleaved by ADAM17 as well [50].

### Cancer

ADAM family has been proved to be key regulators of cell signaling pathway in the tumor microenvironment and ADAM17 was widely involved in tumorigenesis and tumor progression [51–53].

High expression of ADAM17 was confirmed to be related to more secretion of TGF-α and poor prognosis in breast cancer. In addition, increased TGFα and VEGF were seen in MDA-MB-231 breast cancer cells and thus influenced cell proliferation, invasion and angiogenesis [51]. One of the substrates of ADAM17, nectin-4, was easier to detect in breast cancer patients with metastasis [54]. *In vitro* studies indicated the enhancement of ADAM17 in cell proliferation, invasion and metastasis through the activation of PI3K-AKT signaling pathway [51]. In glioma, ADAM17 promoted brain tumor growth, invasion, metastasis, and contribute to stoke-induced neurogenesis [51]. In colorectal cancer, the activation of epidermal growth factor receptors (EGF-R) included the processing of membrane-bound EGF-R which induced by ADAM17. Inhibition of serine hydrolase monoacylglycerol lipase (MAGL) and transcription factor SATB2 could reduce tumor burden of colorectal cancer and was correlated with the downregulation of fibroblast growth factor-2 (FGF-2) and vascular endothelial growth factor (VEGF) [55, 56]. However, the VEGF expression and cleavage of the VEGF-receptor (VEGF-R2) were regulated by ADAM17 [43]. Tyrosine phosphorylation activate ADMA17 and lead to the angiotensin II-induced shedding of HB-EGF [57].

TNFα signaling pathway plays an important role in the tumorigenesis through the regulation of cell apoptosis, death, and survival [4]. Decreased cell membrane receptor-bound TNFα was detected in gastric cancer cells transfected with ADAM17-shRNA. P65, an anti-apoptosis regulator, one of the molecules downstream TNFα, was reduced once ADAM17 downregulated, suggesting that ADAM17 promoted the development of gastric cancer through the regulation of TNFα signaling pathway [58]. Other studies showed that autonomous TNF-α-NF-κB and IL-6-STAT3 signaling are essential for tumor growth while ADAM17 turn on the signaling cascade by the shedding of TNFα [59].

The soluble (s)IL-6R-dependent trans-signaling mediated by ADAM17 was verified as one of the reasons for the development of many cancers including lung cancer, ovarian cancer, pancreatic cancer, colorectal cancer and hepatocellular cancer [60, 61]. In ovarian cancer patients, sIL-6Rα increased in malignant ascites and associated with poor prognosis. IL-6 trans-signaling could influence the chemotherapy-induced apoptosis of endothelial cells and promote the migration of ovarian cancer cells [62]. Moreover, IL-6 could facilitate the progression of pancreatic cancer, while specific inhibitor of IL-6 trans-signaling by the gp130Fc protein could depress tumor growth and further decreased the microvessel density, reduced the number of distant metastases [60]. In hepatocellular cancer, HCC progenitor cells acquired autocrine IL-6 signaling and thus promote the tumor proliferation. Meanwhile, IL-6 trans-signaling was proved to be associate with the gender difference in the incidence of HCC [61]. All these studies suggested that ADAM17-mediated cleavage of the IL-6R is responsible for the tumorigeneses and progression of cancer.

### Central nervous system diseases

ADAM proteases were crucial in the development and regulation of central nervous system, especially axonal growth and myelination [63]. The expression of ADAM17 was higher in fetal brain tissue than adult brain tissue, suggesting the significant role of ADAM17 in the development of neuronal [3]. ADAM17 was detected in neurons and astrocytes, oligodendrocytes, and microglial cells. Cellular localization analysis found that ADAM17-positive neurons often co-localized with amyloid plaques, supporting its role in the pathological process of Aβ formation [64]. ADAM17-mediated cleavage of immunoglobulin superfamily recognition molecule L1 was correlated with the cellular migration and neurite outgrowth [68]. Alzheimer's amyloid precursor protein (APP), which functioned in mediating neuronal migration and synaptic connectivity was verified as substrate of ADAM17 as well [65]. Four and Half LIM domain 2 protein (FHL2), one of the LIM domain proteins, involved in numerous protein-protein interaction and responsible for the generation of soluble and non-amyloidogenic fragment (sAPPα). FHL2 could bind to ADAM17 and co-localized with actin-based cytoskeleton. However, less ADAM17 was detected at the surface of macrophages in FHL2 loss-of-function mice, suggesting that the expression of FHL2 could regulate ADAM17 and participate in neurogenesis [66].

On the other hand, ADAM17 play a role in synaptic plasticity. Neuronal pentraxin receptor, enriched at excitatory synapses, could be cleaved by ADAM17 and release the pentraxin domain, which was necessary for the mGluR1/5-dependent long-term depression in hippocampus and complex coordination in both synapse strengthening and weakening [67]. ADAM17 is important for the synaptic connection formation as well. The processing of RA175/SynCAM1, one cell adhesion molecule involved in the formation of functional synapse, could be inhibited by TNF-alpha protease inhibitor-1 (TAPI-1). Furthermore, the colocalization of RA175/SynCAM1 and synaptophysin on dendrites was increased once TNF-alpha protease was blocked [68].

Other substrates of ADAM17 such as EGF-R showed significant effect on central nervous system and was detected in many types of nerve cells including cerebral cortical pyramidal cells, hippocampal pyramidal cells, Purkinje cells, anterior horn cells, and dorsal root ganglion neurons. Thus, EGF-R signaling which was important to the development of neuronal and synaptic plasticity could be influenced by ADAM17 [69]. As to heparin-binding (HB)-EGF, studies confirmed that it could stimulate the proliferation of CNS astrocytes and multipotent progenitors [70]. Moreover, HB-EGF was responsible for the developing of dopaminergic neurons of the ventral midbrain [71].

Taken together, these findings indicate the importance of the ADAM17 in the development and maintenance of neurons through ectodomain shedding of central nervous system-related membrane-bound proteins.

## ADAM17-related targeted therapy

ADAM17 has been reported to be a regulator of many cellular events and be responsible for the cleavage of growth factors, receptors and adhesion molecules [1, 72]. Human diseases including inflammatory, immune, degenerative diseases and cancer were confirmed to be related to ADAM17. As a result, ADAM17-related targeted therapy is becoming the research hotspot and many of the new potential therapeutic agents have entered into the clinic [73, 74].

**Figure 2:**
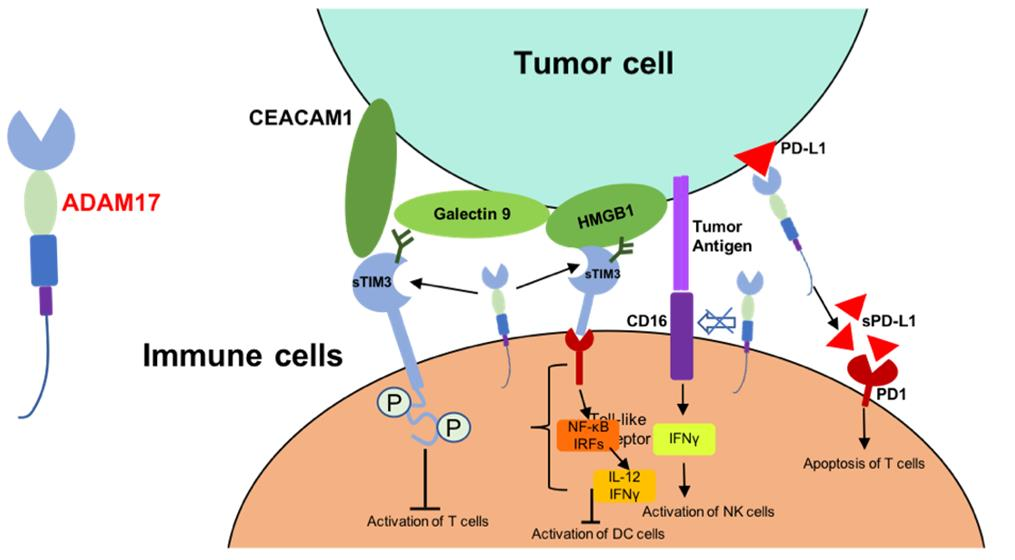
**Mechanism of ADAM17-mediated reduced antitumor activity of immune cells.** Abbreviations: CEACAM1, carcinoembryonic antigen-related cell adhesion molecule 1; HMGB1, high mobility group protein B1; IRFs, interferon regulatory factors; sTIM3, soluble TIM3; sPD-L1, soluble PD-L1.

T cell immunoglobulin and mucin domain-containing protein 3 (TIM3) was one of the cell surface receptors of CD4+ CD8+ T cells [75, 76]. TIM3 was identified as substrates of ADAM17, and soluble TIM3 could bind to carcinoembryonic antigen-related cell adhesion molecule 1 (CEACAM1), galectin9 and high mobility group protein B1 (HMGB1), and thus lead to reduced antitumor activity of immune cells [75, 77, 78]. On the other hand, shedding of membrane-bound CD16 by ADMA17 decrease the expression of IFNγ and other cytokines which are necessary for the activation of immune cells [73]. ADAM17 was associated with the increased soluble PD-L1 from tumor cells. This regulation of ADAM17 induce the apoptosis of T cells and compromise the killing effect of CD8+ T cells [74] (Figure 2). All these suggested that inhibitors of ADAM17 and may be correlated to the tumor targeted therapy selection.

Nowadays, some of the ADAM17 inhibitors were conducted into deep study or entered clinical trials. Small molecules drug which was a dual inhibitor of ADAM17 and ADAM10 could suppress tumor growth and recover the sensitivity of breast cancer cells to EGFR inhibitor [79]. Antibody inhibitors binding both catalytic and noncatalytic domains of ADAM17 showed inhibition of the shedding of ADAM17 substrates including TNFα, TGFα, AREG, HB-EGF, and tumor necrosis factor receptor 1 (TNFR1) [80]. Prodomain of ADAM17 was also tested as inhibitor of ADAM17, it could inhibit the secretion of TNF-α by 5-7-fold depended on different concentration [81]. A stable form of the ADAM17 prodomain was generated and developed as the endogenous specifically inhibitor of cell-surface ADAM17 which not influence ADAM10. This protein inhibitor was verified to attenuate the disease models of sepsis, rheumatoid arthritis (RA) and inflammatory bowel disease (IBD) and reduce the secretion of TNFα [81]. In addition, tissue inhibitors of metalloproteinases (TIMPs) are the endogenous inhibitors of the matrix metalloproteinases, while TIMP3 could bind to the extracellular matrix. Thus, exogenously synthesized inhibitors focus on extracellular domain of ADAM17 may be promising therapeutic agents.

However, challenges such as adverse effect and non-specific inhibition to other closest isozyme (ADAM10) are obstacles we need to resolve [73]. Of note, as ADAMs family are Zn2+- dependent proteases, most of the ADAM inhibitors directly target the active site zinc of zinc-binding moieties of ADAM17, it is hard to avoid off-target effects currently [30]. Multiple substrates of ADAM17 was another factor lead to the side effects of targeted therapy. Inhibitors targeting only a subset of substrates which are more specific is the research direction in the future.

## Regulation network of miRNAs and ADAM17

Accumulating evidence suggested that ADAM17 was up-regulated in various cancers and was involved in tumor growth, invasion and metastasis. However, miRNAs function as upstream regulators in ADAM17 cell signaling pathway and more and more studies focus on the regulation network of cancer-related miRNAs and ADAM family (Table 1) [82–87].

**Table 1:** The regulation network of miRNAs and ADAM17 in different human diseases.

**miRNAs targeted ADAM17**	**Regulation network**	**Pathology conditions**	**Reference**
miR-152	miRNA↑, ADAM17↓	NSCLC	Su et al. 2014
miR-338	miRNA ↓, ADAM17↑	NSCLC Neuroblastoma. Gastric Cancer. HCC	Chen et al. 2013; Hong et al. 2020; Sun et al. 2015; Wang et al. 2015
miR-122	miRNA ↓, ADAM17↑	HCC	Li et al. 2012; Jopling 2012; Thakral et al. 2015
miR-708	miRNA ↓, ADAM17↑	Idiopathic Pulmonary Fibrosis	Liu et al. 2018
miR-143, miR-145 miR-148a, miR-152	miRNA ↓, ADAM17↑	Colon Cancer	Dougherty et al. 2020
miR-326	miRNA↑, ADAM17↓	Hashimoto‘s Thyroiditis	Liu et al. 2020
miR-148	miRNA ↓, ADAM17↑	Nasopharyngeal Carcinoma	Shi et al. 2020
miR-145	Feedback Loop	Renal Cell Carcinoma	Doberstein et al. 2013

Some miRNAs are mainly expressed as tumor suppressors, and ADAM17 were proved to be the direct target of miRNAs [82, 85]. In non-small cell lung cancer (miR-338-3p was down-regulated [83–85, 88]. However, miRNA-mediated overexpression of ADAM17 and activation of downstream substrates lead to the attenuation of suppressive function of miRNA [83–85]. Other tumor suppressor miR-152 showed an inversely correlation with the expression of ADAM17. miR-152-induced tumor suppression effect was partially mediated by down-regulation of ADAM17 expression [89]. In addition, positive and negative feedback loops have been described for ADAM and miRNA in cancer. High level of miR-145 decrease the expression of ADAM17, whereas ADAM17 negatively regulates miR-145 through cleaved substrates such as TNFα [82]. Increased TNFα in tumor microenvironment was responsible for more metastasis. Inhibitors of ADAM family were promising options for cancer treatment through reduction of ADAM substrates and increased tumor suppressive miRNAs in tumor microenvironment [90, 91].

On the other hand, some miRNAs are closely related to the tumorigeneses and development of cancer which may responsible to the target genes and regulation network. miR-122 is a relative tissue specific miRNA which abundantly expressed in liver [92]. It is essential for the metabolism of cholesterol, glucose, lipid and iron homeostasis and it was the first miRNA carry out clinical trial in HCV infected patients [93]. Results from our research group confirmed the function of miR-122 in the regulation of gluconeogenesis and lipid metabolism in HepG2 cells [94]. Moreover, miR-122 was downregulated in HCC and correlated with more aggressive tumor behavior [95]. Targeted transportation of miR-122 to cancer cells using viral vector or liposomal nanoparticles resulted in tumor suppression in HCC animal models [93]. Circulating miR-122 was becoming a prognostic marker in patients with HCC [96]. Additionally, the mechanism that miR-122 involved in the regulation of a large number of target mRNAs has been explored as well. Microarray analysis and 3’-UTR synthetic miR-122 were used to identify the targets of miR-122, whereas CAT-1, ADAM17, BCL-w and interferon-inducible double-stranded RNA-dependent activator (PRKRA) were confirmed [93]. Two independent expression microarray datasets analysis identified 32 candidate target genes of miR-122, most of which were enriched in the cell-cell signaling and gene transcription. ADAM17, one of the target genes of miR-122, was crucial in metastasis. The invasion, migration and angiogenesis were reduced in HCC mice model once ADAM17 was knocked down. miR-122 reduced angiogenesis, inhibit intrahepatic metastasis and functioned as tumor suppressor through the regulation of ADAM17 [97]. Overexpression of ADAM17 was also seen in other cancers including breast cancer, brain tumor and colorectal cancer [98–100]. Of note, ADAM17 was proved to be associated with invasion and metastasis either [98–100].

In other diseases, ADAM17 participated in idiopathic pulmonary fibrosis (IPF) via miR-708-3p/ADAM17/STAT3 signaling pathway. miR-708-3p/ADAM17 axis aggravated IPF and miR-708-3p was verified concentrate in the lungs of animal models [101]. However, in Hashimoto's Thyroiditis (HT), the expression of miR-326 was closely related to the occurrence of HT via the regulation of TH17 cells differentiation [102].

All these results suggested that miRNAs functioned as the upstream regulators of ADAM17 and the miRNA–ADAM17 link participated in various pathological process and diseases.

## CONCLUSIONS

In this review, the functions of different domains and post-translational modifications of ADAM17 have been performed. The links of ADAM17 with different physiological and pathophysiological processes and human diseases are well discussed. As the important role of ADAMs in transcriptional regulation and maintenance of homeostasis, we analysis the regulation network of ADAM17 and miRNAs. The ADAM17 is not simply the target gene of miRNAs, the dual regulation and feedback loops between ADAM17 and miRNAs are also needed to be further understand. All these may contribute to the understanding of ADAM17 and thus provide new insights on the development of more selective ADAM inhibitors and reagents of targeted therapy.
